# Exploration of the visual streak of the Mongolian gerbil as a model for the human central retina

**DOI:** 10.3389/fmed.2025.1562437

**Published:** 2025-04-22

**Authors:** Alexander Günter, Mohamed Ali Jarboui, Regine Mühlfriedel, Mathias W. Seeliger

**Affiliations:** ^1^Division of Ocular Neurodegeneration, Institute for Ophthalmic Research, Centre for Ophthalmology, University of Tübingen, Tübingen, Germany; ^2^Core Facility for Medical Bioanalytics (CFMB), University of Tübingen, Tübingen, Germany

**Keywords:** animal models, human central retina, macular degeneration, Mongolian gerbil, retinal pigment epithelium, visual streak

## Abstract

**Introduction:**

The Mongolian gerbil (MG), a day-active rodent, features a particular retinal region of high visual acuity, the visual streak (VS). Optimized for vision in desert-like environments, the VS allows for a perfect view of the horizon between the projection areas of the sky and the ground. Here, we assess the structural basis of this specialized region and compare the findings to the conditions at the human retinal center.

**Methods:**

The VSs of MG retinas (*n* = 5) were evaluated morphologically with immunohistochemistry for cone, rod, and RPE cell-specific markers in dorsoventral cross-sections, and the results were compared to data from the near (adjacent) and far periphery. Mass spectrometry of the VS and peripheral retina/RPE was used to analyze the proteomic differential expression between these regions.

**Results:**

In the VS of the MG, we found an increased density of cones, elongated photoreceptor outer segments (OSs), and a rod-to-cone ratio lying within the zone of descent between the border of the macula and the fovea (macular shoulder). Similarly, the base area of retinal pigment epithelium (RPE) cells in the VS was significantly reduced, while cells were taller than those in the periphery. Accordingly, proteomic data provided evidence for an enhanced abundance of key proteins relevant to photoreceptor and RPE function and pathophysiology of macular diseases in the VS.

**Conclusion:**

The high degree of conformance between the VS data of the MG and the human central retina renders the MG a promising rodent, non-primate model of the central human retina.

## 1 Introduction

Human reading and color vision very much depend on the function of the cone photoreceptor system in the central retina at the back of the eye, in particular, the macula. Inherited and acquired diseases that lead to general or localized dysfunction of these sites impair regular vision up to legal blindness (1). The most commonly known disease is age-related macular degeneration (AMD), the leading cause of irreversible blindness in individuals over 50 years of age. It is a chronic and progressive disease characterized by the degeneration of photoreceptors and retinal pigment epithelium (RPE) cells in the macula. In AMD, slow degeneration of the RPE cell layer and photoreceptors due to the accumulation of extracellular deposits (drusen and subretinal drusenoid deposits) are hallmarks of the disease (2, 3). To investigate retinal physiology and pathophysiology and to develop therapeutic strategies, models that have a high conformance with the human situation are needed. Unfortunately, mice are currently the most common models in terms of genetic manipulation to produce homologous disease genotypes but are—due to their nocturnal lifestyle—physiologically not optimally suited to study diseases of the central retina. In this study, we have assessed whether the Mongolian gerbil (MG), a diurnal rodent originating from an environment of semi-deserts and steppes (4), holds retinal properties useful in further research on human central retinal disease.

The mammalian retina uses rod and cone photoreceptors as primary sensory cells to detect light. Rods and cones translate light stimuli into electrical signals, which travel along the retinal network to the brain (5). Rods are most sensitive to light stimuli in dim light (scotopic) conditions, while cones provide high-acuity and color vision in brighter (photopic) conditions. Photoreceptors are supported by the RPE, a monolayer of cells that forms the outer blood–retinal barrier (6). The RPE provides important support to photoreceptors, including regulation of vitamin A supply, absorption of excess light by melanin, and participation in the daily renewal of ROS. RPE dysfunction may thus lead to retinal degeneration (7).

The human central retina is a specialized region for high-acuity vision with a diameter up to 6 mm (8, 9). At the center of this region, the fovea is located, featuring the highest visual acuity. It is densely packed with cones having elongated outer segments (OSs) but contains no rods. Adjacent to the fovea, para- and perifovea are present, which are ring-shaped regions characterized by a continuous increase in rod and a decrease in cone cell density with eccentricity that form the ‘macular shoulder’. The supporting RPE cells in the central retina show a reduced base area, but they are taller when compared to more peripheral regions (10, 11). To further improve visual performance, there are no retinal vessels intersecting the path of light in the central retina to avoid any interference with vision (8, 12–14).

**Figure 1:**
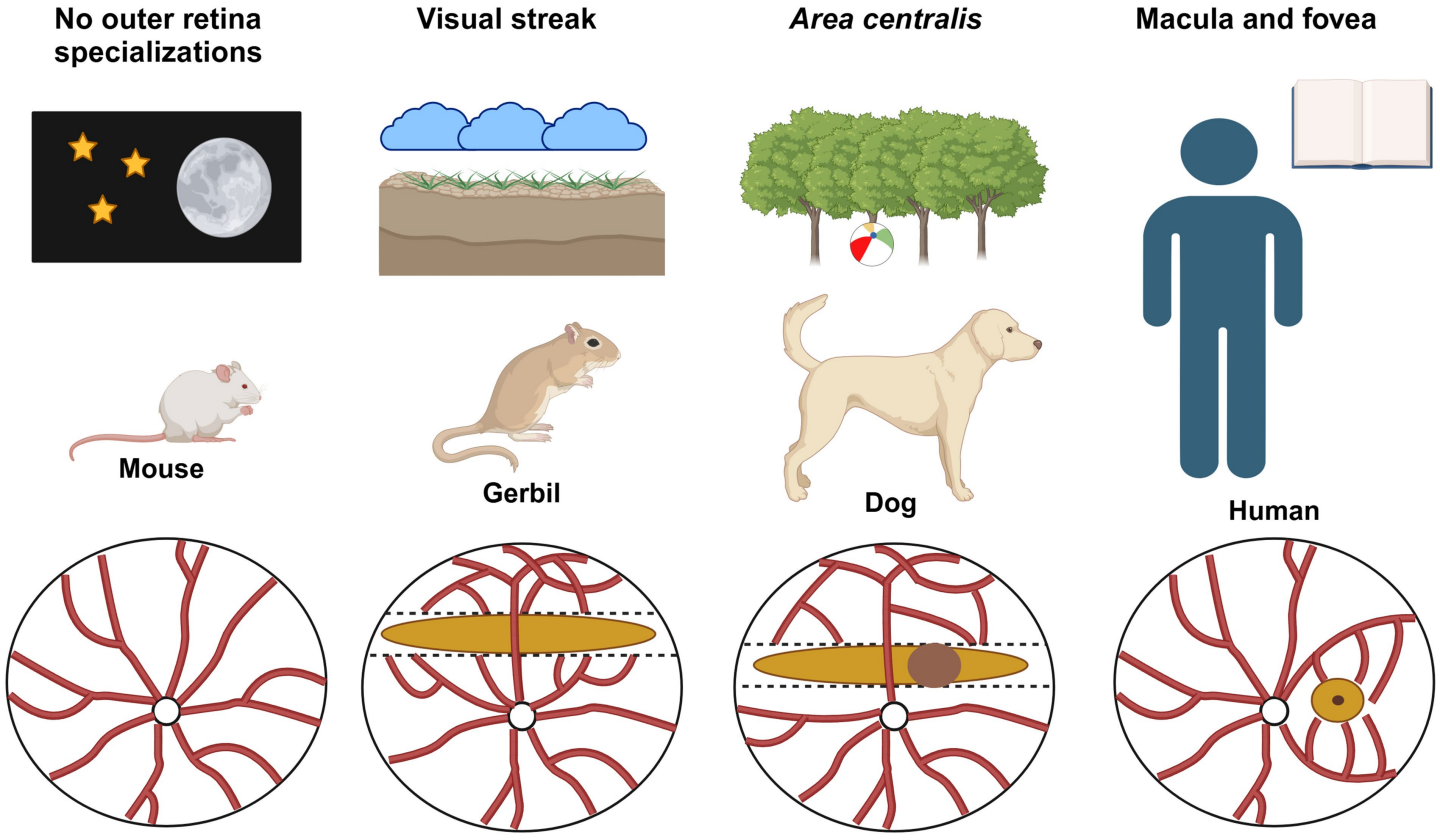
Examples of different retinal organizations. Mice are nocturnal animals and, therefore, require a high degree of light sensitivity (provided by rods) but no site for high-acuity vision (provided by cones). The absence of a clearly recognizable high-acuity region in the outer retina may be observed as a rather homogeneous build-up of the murine retina in terms of the vasculature and rod/cone distribution (left). Diurnal rodents such as the Mongolian gerbil (MG) originate from the semi-deserts and steppes of central Asia and use a visual streak (VS) to scan the horizon for potential predators and their flock. To enhance image quality, intersecting blood vessels that would optically interfere are reduced to a minimum (second to the left). Some predators, such as dogs and cats, have an additional ring-shaped area of high visual acuity within or close to the VS, the area centralis. These areas allow for some binocular vision that cannot be obtained by the superposition of VSs. Humans heavily rely on binocular vision and have a very high-resolution area, the macula, with a central rod-free fovea. White circle: optic nerve head; red lines: major retinal vessels; dark yellow bar: VS; dark brown circle: area centralis; dark yellow circle with black dot: macula with central fovea. These regions represent higher cell densities. Created in BioRender. Mühlfriedel, R. (2025) https://BioRender.com/y12g151.

The retinal organization of species often arises from their habitat and role in the animal community (e.g., predator vs. prey). The evolutionary pressure to effectively collect the visual information that fits the specific need for survival and reproduction may lead to major differences in retinal topography (15, 16). Mice live in a nocturnal environment and lack a clearly recognizable region of high-acuity vision throughout the outer retina (17). In the inner retina, shallow type-specific ganglion cell gradients have been documented (18). Humans, on the other hand, are diurnal and rely on binocular vision that stimulates the development of a very advanced cone system organization (i.e., macula and fovea). In many mammalian species, a horizontal VS is found, which is ideal for habitats with visual scenes dominated by the horizon and a clear separation of sky and ground. Predators with a higher demand for acuity often develop an additional area centralis within or adjacent to the VS (19, 20). Examples of these different retinal organization types are given in Figure 1.

Mice are currently the most widely used animal models for research on retinal diseases. While major advances in understanding rod-based disease mechanisms have been feasible, research on cone-based disorders has been limited by the anatomically and physiologically aberrant cone system when compared to humans. Although the fraction of cones within all photoreceptors (approximately 3–5%) is similar to the human ratio, their build-up and topographical organization are very different, as shown above. Most importantly, the majority of cones in mice co-express the cone-specific optical pigments S-and M-opsin, which follow counter-directional dorsoventral gradients (21–23). In contrast, there is only one type of opsin per cone expressed in both human and MG retinas, and the expressions of different opsins do not follow a similar gradient (24). Although the VS of the MG is not a rod-free region like the very central fovea, it nevertheless shares important characteristics with the macular shoulder, a region outside the fovea but within the central retina. The macular shoulder has been identified as an area where initial pathological changes are found in a number of disorders, including AMD, so an animal model of this area would be of great relevance (25, 26). For these reasons, it was investigated in this study whether the MG may satisfy the high unmet need for small animal models with a cone system organization that better matches the human situation.

## 2 Materials and methods

### 2.1 Experimental animals

All animal experiments and procedures performed in this study adhered to the Association for Research in Vision and Ophthalmology (ARVO) statement for the Use of Animals in Ophthalmic and Vision Research and were approved by the competent legal authority (Regierungspräsidium Tübingen, Germany). All efforts were made to minimize the number of animals used and their suffering. MGs were housed under an alternating 12-h light and dark cycle, with free access to food and water, and were used irrespective of gender. Five adult MGs aged 2–3 months were used for immunohistochemical experiments, and two MGs aged 2 years were used for proteomics analysis.

### 2.2 Immunohistochemistry

MGs were euthanized via CO_2_ administration. The eyes received a temporal burn mark at the cornea, were enucleated, and a small cut was made at the retinal eyecup adjacent to the corneal mark for the orientation of the tissue. Eyes were cut along the ora serrata to remove the cornea, lens, and vitreous and were fixed in 4% w/v paraformaldehyde (PFA) for 45 min. After that, they were immersed in 30% w/v sucrose phosphate buffer (pH 7.4) overnight at 4°C. The next day, eyes were embedded in Tissue-Tek optimal cutting temperature (OCT) compound (Sakura Finetek Europe, Alphen aan Den Rijn, Netherlands), slowly frozen using dry ice, and then stored at −80°C. Retinal dorsoventral cross-sections with a 12-μm thickness were collected on Superfrost glass slides (R. Langenbrinck GmbH, SuperFrost^®^ plus, Emmendingen, Germany) and stored at −20°C. For the immunohistochemical staining process, slides were dried at 37°C for 30 min and rehydrated with phosphate-buffered saline (PBS) for 10 min at room temperature (RT). As a next step, they were incubated for 1 h at RT with a blocking solution that consisted of 5% v/v chemiBLOCKER (Merck, Darmstadt, Germany) in PBS containing 0.1% v/v Triton X-100. Afterwards, the sections were incubated overnight at 4°C with the following antibodies diluted in the blocking solution: fluorescein isothiocyanate (FITC)-conjugated peanut agglutinin (PNA) (L7381; 1:100; Sigma-Aldrich, St. Louis, MO, USA), anti-rhodopsin (ab98887; 1:500; Abcam, Berlin, Germany), and anti-MERTK (ab95925; 1:100; Abcam). The use of detergent in the blocking solution allowed for the visualization of intracellular MERTK + and rhodopsin + labeled phagosomes. The next day, slides were washed 3x in a washing solution containing PBS with 2% v/v chemiBLOCKER at RT. Then, the following secondary antibodies diluted in the washing solution were incubated for 2 h at RT: Goat anti-Rabbit, Alexa Fluor 568 (A11036; 1:300; Thermo Fisher Scientific, Karlsruhe, Germany) and Goat anti-Mouse, Alexa Fluor 647 (ab150119; 1:150; Abcam). As a last step, sections were rinsed with PBS and mounted with ROTI Mount FluorCare containing DAPI (4′,6-diamidino-2-phenylindole) (Carl Roth, Karlsruhe, Germany). DAPI was used to identify rod and RPE nuclei, and MERTK was used as a marker for RPE cells.

### 2.3 Microscopy and image analysis

Sections (12 μm thickness) used for immunohistochemistry were imaged on a Zeiss Imager Z.2 fluorescence microscope equipped with ApoTome 2, an Axiocam 506 mono camera, and an HXP-120 V fluorescent lamp (Carl Zeiss Microscopy, Oberkochen, Germany). Z-stack images (12-bit depth, 1,384 * 1,040 pixels, pixel size = 0.323 μm, Z-planes at 1 μm steps) were captured with the ZEN 3.3 (blue edition) software (Carl Zeiss Microscopy) with a 20x air objective (N.A. 0.8). The quantification of photoreceptor densities, cone OS length, and number of phagosomes/RPE cell was done by averaging measurements from at least four sections per animal in 1,200 μm^2^ sections (100 μm dorsoventral segments x 12 μm z dimension thickness) from the VS, the near, and the far peripheral retina. OSs labeled with PNA were manually counted through the Z-stack to determine the density of cones. The length of the cone OSs and RPE height were determined as an average of three distinct single measurements per section. To determine the density of rods, the average diameter of the outer nuclear layer (ONL) nuclei and the number of ONL rows arising from rod cells were quantified. These values were used to calculate the number of rods in 100 μm dorsoventral sections. Since the retinal sections presented a thickness of 12 μm (z dimension), the average ONL nuclei diameter was used to calculate the number of rod cells in the z dimension. After multiplying these values with each other, the rod density in 1200 μm^2^ was obtained. Finally, the photoreceptor densities calculated in 1200 μm^2^ were estimated for an area of 1 mm^2^. To determine the number of phagosomes/RPE cells, phagocytized OS tips that were MERTK + and rhodopsin + were manually counted through the Z-stack and divided by the number of RPE cells present in that section. The VS was identified in the dorsoventral cross-sections as a region in the dorsal mid-peripheral portion of the retina that is characterized by a thicker inner retina (visualized with DAPI) and elongated OSs, which were well visible when stained with a cone OS marker such as PNA. Dorsal and ventral sections taken approximately 300 μm from the center of the VS were considered as near peripheral retina. Dorsal sections taken approximately 2 mm from the center of the VS were considered far periphery, as well as ventral sections with the same contralateral position. Preference was given to sections with a strong adherence between the retina and RPE and no artificial fragmentation of the OSs. Figures were prepared using Adobe Photoshop CS5 (Adobe, San Jose, CA, United States) and the CorelDRAW X5 software (Corel Corporation, Ottawa, ON, Canada).

### 2.4 Calculation of RPE cell area

For the calculation of RPE cell areas in the VS and the periphery of the MG retina, the distances between the center of RPE cell nuclei were quantified in 250 μm dorsoventral retinal segments. As some RPE cells are binucleate, we had to consider this fact in our calculations. Within the 454 RPE nuclei analyzed in the VS, 64 (14%) were classified as binucleate cells, and in the peripheral retina, the fraction was 73 of 703 (10%). Internuclear distances that were below half the median of the VS data (17.12 μm) or of that of the peripheral retina (20.57 μm) were considered artifacts arising from binucleate RPE cells, and distances that were above twice the medians were considered artifacts from sections where a correct intermediate nucleus was missing (4% for the VS and 5% for the peripheral retina) (Supplementary Figure 1C). For binucleate RPEs, the center between the nuclei was used to calculate the distance to the next RPE cell nucleus. The quantification of distances between RPE nuclei from the above-corrected data is shown in Supplementary Figure 1A.

As a next step, the RPE internuclear distances were used to calculate the resulting RPE cell areas. In detail, the calculation uses two basic formulas for equilateral triangles of side length *a* (see the sketch in Supplementary Figure 1B):


$$\mathrm{Height}\;h=\frac{\sqrt{3}}{2}\times a$$

$$\mathrm{If}\;\mathrm{the}\;\mathrm{internuclear}\;\mathrm{distance}\;D=2\times h=\sqrt{3}\times a,\mathrm{then}\;a=\frac{D}{\sqrt{3}}$$

$$\mathrm{Area}\;\mathrm{of}\;\mathrm{an}\;RPE\;\mathrm{hexagon}\;A=3\times h\times a=3\times \sqrt{3}\times \frac{a^{2}}{2}$$

$$A=3\times \sqrt{3}\times \frac{D^{2}}{3\times 2}=\frac{\sqrt{3}}{2}\times D^{2}$$

### 2.5 Mass spectrometry

Eyes from MGs were enucleated, and the VS was isolated with a scalpel under a dissection microscope. The VS appears as a light horizontal band visible in the retina. Accordingly, a dorsal region adjacent to the VS was isolated and was the same size as the former one. Tissue from these regions was sent for mass spectrometry as (1) RPE + retina combined or (2) as isolated RPE, which was mechanically separated from the retina in a phosphate buffer (PB) solution. Both tissue preparations (1) and (2) also contained elements of the choroid and sclera. All tissues were put in liquid nitrogen immediately after preparation and stored at −80°C. The analysis was performed on an Ultimate3000 RSLC system coupled to an Orbitrap Tribrid Fusion mass spectrometer (Thermo Fisher Scientific), as previously described (27).

Spectra were acquired in data-independent acquisition (DIA) mode using 50 variable-width windows over the mass range of 350–1,500 m/z. The Orbitrap was used for MS1 and MS2 detections, with an AGC target for MS1 set to 20×10^4^ and a maximum injection time of 100 ms. The MS2 scan range was set between 200 and 2000 m/z, with a minimum of 6 points across the peak. Orbitrap resolution for MS2 was set to 30 K, the isolation window was set to 1.6, the AGC target was set to 50×10^4^, and the maximum injection time was set to 54 ms. MS1 and MS2 data were acquired in centroid mode. To reduce the possibility of carry-over and cross-contamination between the samples, one TRAP and two BSA washes were used between sample groups. We used quantification based on MS2 spectra, which is a superior method to the standard data-dependent acquisition relying on MS1 quantification.

### 2.6 Statistical analysis

For the immunohistochemical data, two-tailed paired Student’s t-tests (two groups) and a repeated-measures one-way ANOVA (three groups), followed by a Bonferroni correction for multiple comparisons with a confidence interval of 95%, were used to assess statistical differences between quantitative data from two and three groups, respectively. The Shapiro–Wilk normality test was used to support the assumption of normality of the data, and the homogeneity of variance (sphericity) for the repeated-measures one-way ANOVA was sufficient according to the Geisser–Greenhouse correction. All analyses were based on five individual animals. Values of *p* < 0.05 were considered to be statistically significant and labeled with an asterisk (*) in the graphs. To indicate a higher degree of statistical significance, values of *p* < 0.01 were marked with two (**), *p* < 0.001 with three (***), and *p* < 0.0001 with four (****) asterisks. Graphical results are represented as mean ± standard deviation for photoreceptor density, OS length, normalized RPE height, and number of phagosomes/RPE cell metrics. In boxplots, used to display ratios, the distance between RPE cell nuclei, and RPE cell areas, the box limits represent the 25–75% quantile range, the central line represents the median, and the whiskers designate the 5 and 95% quantiles. The statistical analysis was performed with GraphPad Prism 10.1 for Windows (GraphPad Software, La Jolla, CA, United States).

Mass spectometry raw data were analyzed using DIA-NN 1.8.2 beta 27 (28) in library-free mode against the NCBI Gerbil database (release December 2023, 21,261 proteins). First, a precursor ion library was generated using FASTA digest for library-free search in combination with deep learning-based spectra prediction. An experimental library generated from the DIA-NN search was used for cross-run normalization and mass accuracy correction. Only high-accuracy spectra with a minimum precursor FDR of 0.01 and only tryptic peptides (two missed tryptic cleavages) were used for protein quantification. The match-between-runs option was activated, and no shared spectra were used for protein identification.

Data were further analyzed using the Perseus platform (29). First, data were log2-transformed to facilitate the identification of proportional changes in protein abundance between the different groups. Only proteins identified and quantified in all replicates of at least one biological group were retained for further analysis. This transformation helps normalize the data and stabilize the variance, making it more suitable for subsequent statistical analysis. To determine significant differences in protein abundance between groups, a two-sample t-test was used. This test was complemented by a permutation-based false discovery rate (FDR) control method, set at a threshold of 0.05, to account for multiple comparisons and reduce the likelihood of type I errors. Specifically, 250 random permutations of the data were performed to generate the null distribution and calculate the FDR-adjusted *p*-values. This approach ensures robust identification of statistically significant changes in protein abundance.

## 3 Results

### 3.1 The visual streak of the Mongolian gerbil: cone photoreceptor density, OS length, and rod-to-cone ratio

**Figure 2:**
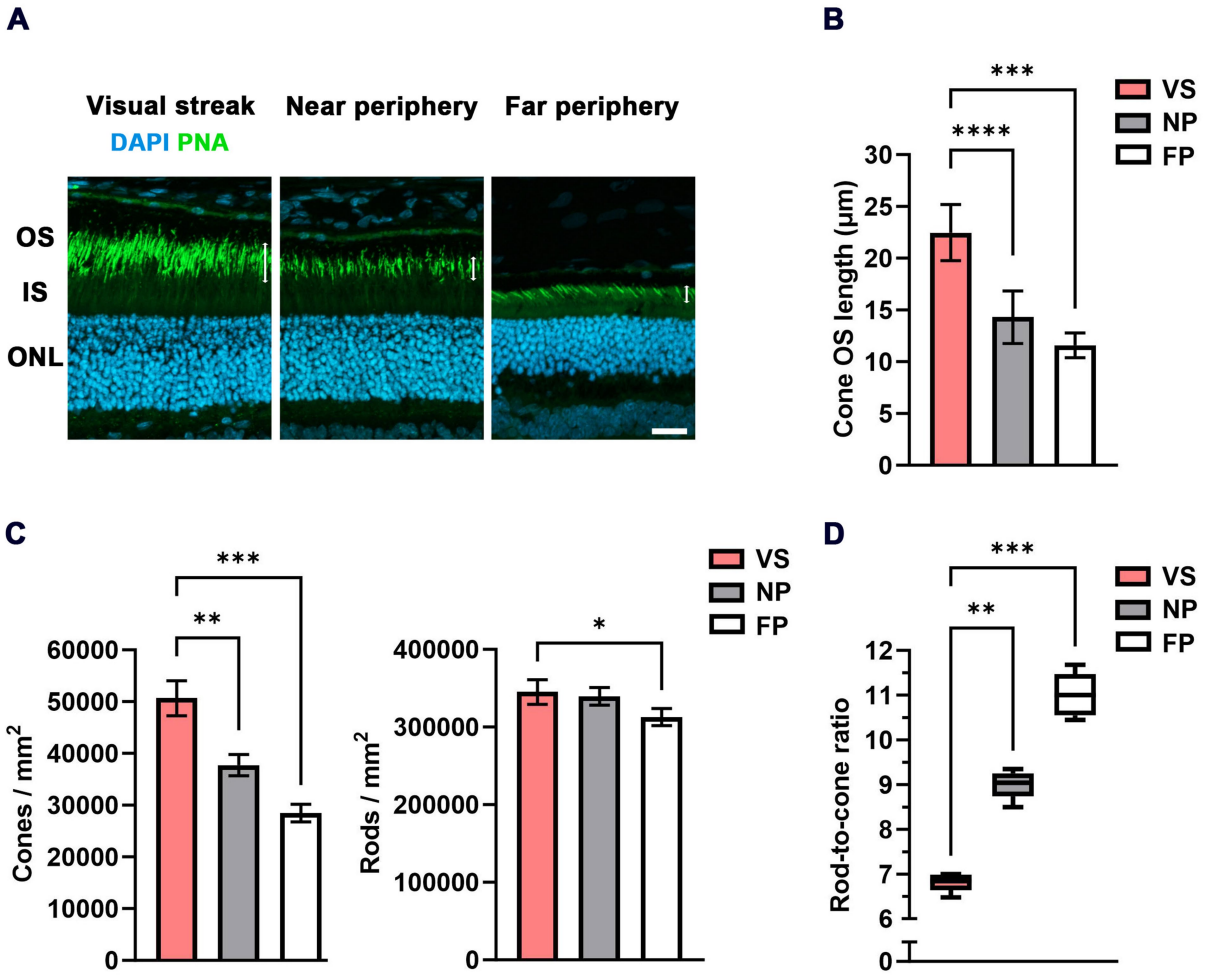
Densities of photoreceptors and RCR in the VS of the MG. **(A)** Cone outer segment (OS) immunohistochemistry (PNA-based) indicates an increase in OS length in the VS when compared to the near and far peripheral regions (white double arrows). DAPI was used for nuclear counterstaining and for counting photoreceptors in the outer nuclear layer (ONL). IS = inner segment; OS = outer segment; scale bar = 20 μm. **(B)** Quantification of the cone OS length in μm in the VS compared to the near and far peripheral retinas (*n* = 5 eyes). Error bars represent the standard deviation. **(C)** Quantification of the density of cones / mm^2^ and rods/mm^2^ in the VS compared to the near and far peripheral retinas (*n* = 5 eyes). Error bars represent the standard deviation. **(D)** Quantitative evaluation of the RCR (box-and-whisker-plot) in the VS compared to the near and far peripheral retinas (*n* = 5 eyes). Boxes: 25–75% quantile range, whiskers: 5 and 95% quantiles, central line: median; * = *p* < 0.05, ** = *p* < 0.01, *** = *p* < 0.001 and **** = *p* < 0.0001. VS = visual streak, NP = near periphery, and FP = far periphery.

A key feature of high-acuity vision is a substantially increased density of cone photoreceptors in respective topographic areas. Our data show that this is also true for the VS of the MG but with a less steep slope than the one observed in its human counterpart (30). We found a cone density of 50,660 ± 3,370 cones/mm^2^ in the VS, much higher than the densities of 37,743 ± 2054 cones/mm^2^ and 28,476 ± 1,696 cones/mm^2^ in the near and far periphery, respectively (Figures 2A,C; Table 1). In contrast, the rod photoreceptor density does not usually vary to a similar degree between the center and periphery, as spatial resolution is less relevant than sensitivity in dim light conditions. We recorded a rod density of 345,014 ± 15,774 rods/mm^2^ in the VS, 339,391 ± 11,337 rods/mm^2^ in the near periphery, and 312,946 ± 10,978 rods/mm^2^ in the far periphery (Figure 2C; Table 1). These distribution characteristics are in agreement with a high-acuity topography of the MG retina, unlike that found in mice (31).

**Table 1:** Comparison of photoreceptor and retinal pigment epithelium (RPE) characteristics of Mongolian gerbils and humans.

Mongolian gerbil (MG)	Rod count per mm^2^ (x1,000)	Cone count per mm^2^ (x1,000)	Rod-to-cone ratio (RCR)	RPE cell area in μm^2^
Visual streak (VS) ^*^	**354** [328–357] (87%)	**51** [47–54] (13%)	**6.84** [6.64–6.98]	**317.9** [294.9–329.1]
Far periphery ^*^	**313** [304–322] (92%)	**29** [27–30] (8%)	**11** [10.55–11.47]	**442.1** [392.5–467.2]

Human	Rod count per mm^2^ (x1,000) ^†^	Cone count per mm^2^ (x1,000)^†^	Rod-to-cone ratio (RCR)^†^	RPE cell area in μm^2 (‡, §)^
Central retina ^#^ (Macular shoulder, at 1.5 mm eccentricity)	**100** (87%)	**15** (13%)	**7**	**177.55** ± 15.69
Far periphery ^#^ (20 mm eccentricity)	**50** (91%)	**5** (9%)	**10**	**331.87** ± 27.23

*Median [25–75% quantiles] (fraction of the total photoreceptor count). †Human photoreceptor data from (9, 30). # (fraction of the total photoreceptor count). ‡Human RPE data from (11). §Median ± SD.

Furthermore, a specific finding in the human central retina is the elongation of the cone OSs (32). In the MG, we observed a comparable elongation of cone OSs (22.47 ± 2.71 μm) in the VS relative to those in the near (14.3 ± 2.53 μm) and far (11.59 ± 1.2 μm) peripheral retinal regions (Figures 2A,B). This similarly applies to rod cells (24) to allow for physical contact with the supporting RPE. A proteomic differential expression analysis between the VS and the peripheral retina/RPE interface was performed to obtain further insight into possible changes in protein regulation. We found that rod OS markers, such as rhodopsin, and cone OS markers, such as M-opsin, were relatively upregulated in the VS, which supports the elongation of cone OSs in our study (Supplementary Figure 2A). A list of all proteins identified and quantified across the VS and periphery is presented in Supplementary Spreadsheet 1.

An important marker amalgamating the distribution topography of rods and cones is the rod-to-cone ratio (RCR). The fraction is formed as rod density may become zero (in the fovea), but cone density does not; therefore, rod density is not suitable as a denominator. This means that the RCR is low in regions of high acuity and high in less specialized areas. In the VS of the MG, we found an RCR of 6.8 ± 0.2, significantly lower than the ratios of 9 ± 0.31 in the near periphery and 11 ± 0.48 in the far periphery (Figure 2D). A comparison with the human data of the macular shoulder area is given in Table 1. We found that both the ratios in the VS of the gerbil (RCR 6.8) and the human central retina (RCR of 7 at 1.5 mm eccentricity), as well as the ratios in the peripheral retina (RCR 11 in the MG) and the far peripheral region of the human retina, were comparable.

### 3.2 The visual streak of the Mongolian gerbil: RPE cell area and shape

**Figure 3:**
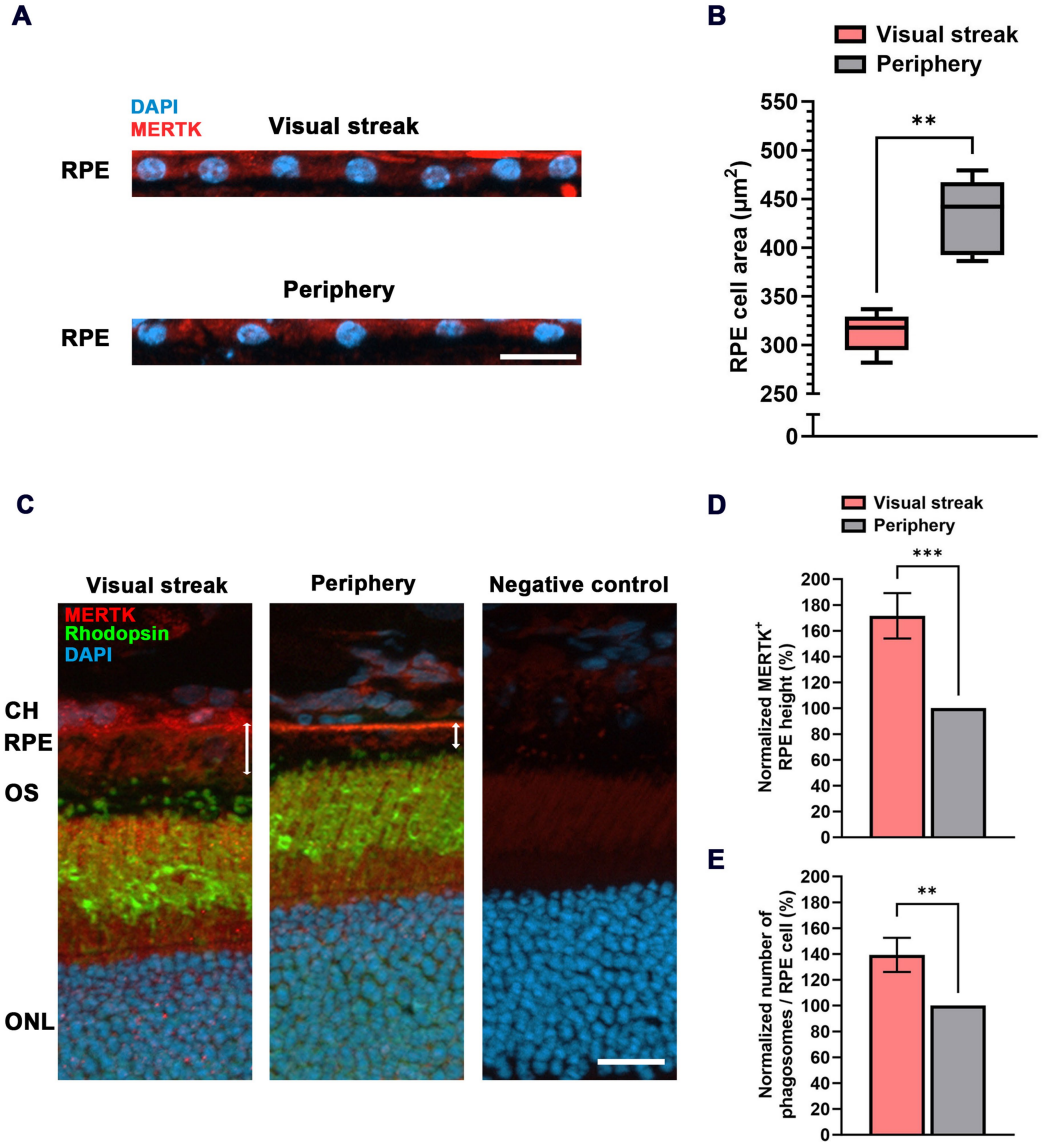
RPE cell characteristics in the VS of the MG. **(A)** Immunohistochemical staining of nuclei (DAPI) in the RPE monolayer of the VS (top) and the peripheral retina (bottom). MERTK was used to mark the RPE layer. RPE = retinal pigment epithelium; scale bar = 20 μm. **(B)** Quantitative evaluation of the cell area in μm^2^ (box-and-whisker-plot) in the VS compared to the peripheral retina (samples from *n* = 5 eyes). Boxes: 25–75% quantile range, whiskers: 5 and 95% quantiles, central line: median. **(C)** Increased height of RPE cells (marked by MERTK staining) in the VS when compared to those in the periphery (white double arrows). CH = choroid; OS = outer segment; ONL = outer nuclear layer; scale bar = 20 μm. **(D)** Quantification of relative RPE height in the VS normalized to the peripheral retina (*n* = 5). **(E)** Quantitative evaluation of the relative number of Phagosomes/RPE cell in the VS normalized to the periphery (*n* = 5). The error bar represents the standard deviation; ** = *p* < 0.01 and *** = *p* < 0.001.

In addition to the conformance between the VS of the MG and the human central retina in terms of photoreceptor morphology and distribution, we moved on to an analysis of the morphology and distribution of the underlying RPE cells. In human studies, it has been observed that the horizontal extensions (and thus the area) of RPE cells in the central retina are smaller than in the periphery (11, 33, 34) (Table 1), while in mice, the opposite gradient is observed, with central RPE cells capturing the largest area in comparison to other subpopulations of RPE cells (31, 35). In the MG, we recorded the distances between RPE cell nuclei as a measure of horizontal extension and calculated the respective average area per cell (Figures 3A,B) based on a formula as shown in the Methods section and Supplementary Figure 1B.

RPE cells form a monolayer that can replace gaps by the division of neighboring cells, so a certain number of RPE cells are known to be binucleated (i.e., having two nuclei). We, therefore, checked the abundance of such binucleate cells in our samples and removed these from the calculations of cell area. The average horizontal extension of RPE cells in the VS of the MG (19.16 ± 0.62 μm) was smaller in comparison to the 22.59 ± 1.01 μm found in the periphery (Figure 3A; Supplementary Figure 1A). The extension data were used to calculate the cell area, resulting in an RPE cell area of 317.9 ± 20.34 μm^2^ in the VS, significantly smaller than the area of 442.1 ± 38.97 μm^2^ in the peripheral retina (Figure 3B).

In addition to the reduced area, human RPE cells in the macula are also taller when compared to those in the periphery (10). We found the same effect in the VS of the MG based on a Mer tyrosine kinase (MERTK) staining, indicating an increase of 71.6 ± 17.5% in height compared to the peripheral retina (Figures 3C,D). This increase in height (~70%) is more than what would be needed to compensate for the reduced area (~30%) in terms of an increase in RPE cell volume, suggesting an increased content of intracellular phagosomes containing material from photoreceptor OS tips (Figure 3C). Indeed, there was an elevation of 39.3 ± 13.2% in the number of Phagosomes/RPE cell in the VS compared to the peripheral retina (Figure 3E). This is supported by a proteomic differential expression analysis between the VS and the peripheral retina/RPE interface, which confirmed an enhanced abundance of MERTK (Supplementary Figure 2A) together with non-membrane-bound RPE proteins that directly interact with MERTK for the engulfment of OS phagosomes such as Gas6 and focal adhesion kinase (FAK) (Supplementary Figure 2B) (36). In line with these findings, OS and RPE proteins related to the visual cycle were also more abundant in the VS of the MG when compared to the peripheral retina (Supplementary Figures 2A,B). Gene expression analysis between the human macula and the periphery showed a higher expression of key OS phagocytosis (37) and visual cycle (38) markers in the macula.

## 4 Discussion

In this study, we assessed the degree of conformance between the VS of the MG and the human central retina as part of the search for a rodent, non-primate model system for central human retinal disorders. Specifically, we characterized photoreceptor density, cone OS length, RCRs, RPE cell extension, height, area, and the number of phagosomes/RPE cell between the VS and the retinal periphery. We found that the maximal RCR in the VS of the MG lies within the RCR range in the human macular shoulder area. Moreover, similar to the human macular shoulder, the RPE cell area in the VS was smaller, and the cells were taller in comparison to the peripheral retina. The increase in height (approximately 70%) exceeded the reduction in area (approximately 30%), indicating an increased volume of central RPE cells. This may explain the enhanced abundance of key proteins for photoreceptor OS phagocytosis and RPE visual cycle in the VS of the MG found in the proteomic data.

**Figure 4:**
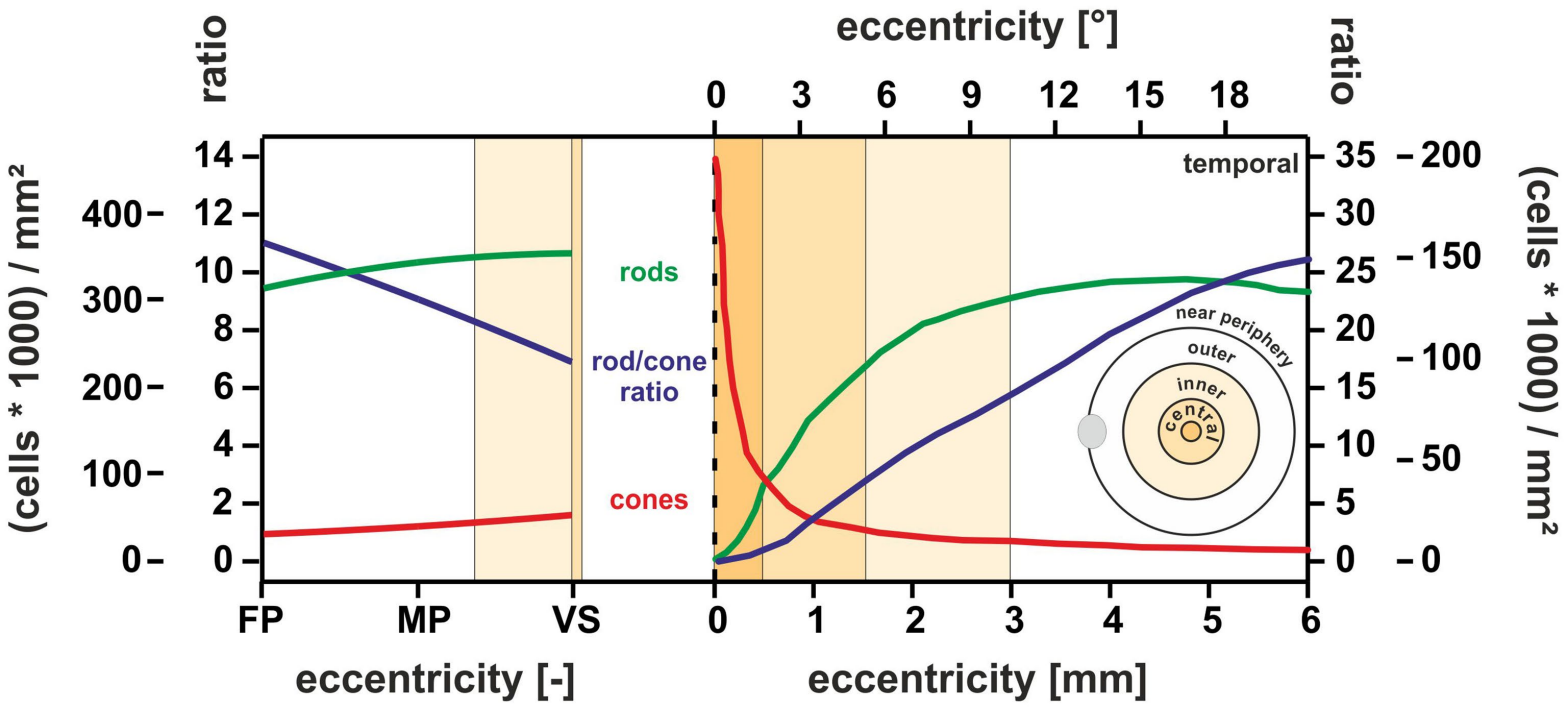
Degree of conformance of retinal photoreceptor densities and RCR between the MG and the human retina. *Left*: MG data for the three eccentricity regions VS, mid-periphery (MP), and far periphery (FP). *Right*: Human eccentricity data redrawn from (9), Figure 2. The courses of rod (green) and cone (red) photoreceptor density (scale (cells*1,000)/mm^2^) and the resulting rod-to-cone ratio RCR (blue) indicate a similar topographic transition characteristics from the periphery (left end in the MG and right end in humans) toward the retinal center, which is characterized by the VS in the MG (right end of the left partial graph) and the macular shoulder/fovea in the human retina (left end of the right partial graph). The VS data match the human situation in the central retina at approximately 1.5–2 mm eccentricity (macular shoulder range).

In terms of research on macular degenerations such as AMD, the macular shoulder has been identified as an important area for understanding disease onset, as this region is particularly vulnerable to age-related changes (25, 39). Before the occurrence of characteristic visible disturbances in the RPE cells, AMD starts in the macular shoulder area with the cell death of mainly rods during the early stages of this disease, together with a severely reduced rod sensitivity in this region (9, 26). Since the VS of the MG showed an RCR within the human macular shoulder range, it appears to be a promising model for future study to understand the pathophysiology of AMD. Beyond that, the overall photoreceptor densities and the resulting RCR of the MG showed a topographic transition from the retinal periphery to the center, as does the human retina (Figure 4). In comparison, mice show the lowest RCR of 29, which does not lie within the range of the human macular shoulder or even the near periphery (9, 31) due to their species lifestyle (15).

To further understand the pathophysiology of macular diseases, a detailed assessment of the interaction of photoreceptors with RPE cells is of great importance. However, this appears to be dependent on a valid representation of macular RPE cells in a model system, as these are different from peripheral ones. To address this aspect, we initially compared the morphological RPE cell features of humans and the MGs. Our results regarding RPE cell height indicate that the human RPE cell characteristics (macular height of 10–15 μm vs. peripheral of approximately 7 μm) (10, 40, 41) were close to those found in the VS (12.08 ± 4.1 μm) and periphery (7 ± 2.1 μm) of the MG. In terms of photoreceptors, the OS length of human cones (22 μm in the macular shoulder vs. 6.5–13 μm in the periphery) (42) is also comparable to the values found in the VS (22.47 ± 2.71 μm) and periphery (11.59 ± 1.2 μm) of the MG.

Additionally, we obtained proteomic profiles of the retina and RPE in the VS of the MG and compared those to data from the peripheral retina. This analysis supports the difference between central and peripheral RPE cells in the MG. In the RPE of the VS, we found an enhanced abundance of MERTK and proteins that directly interact with it (Gas6 and FAK) when compared to the peripheral retina. MERTK is a receptor present on the apical membrane of the RPE that binds indirectly to exposed phosphatidylserine of photoreceptor OSs via a soluble ligand and is necessary for the intracellular engulfment of OS particles (7, 43). Biologically, enhanced MERTK activity is directly related to an increased number of phagosomes/RPE cell as found in the VS, which indicates higher levels of OS phagocytosis in this area. We hypothesize that RPE cells in the VS, due to their increased height, volume, and increased number of phagosomes, together with the increased length of photoreceptor OSs and the elevated cone photoreceptor density, may encounter a higher phagocytic load and thus may be at risk of a pathological accumulation of undigested material as intracellular deposits during rod OS deterioration and cell death, which we believe is a further important asset for a model of macular degeneration. Thus, the assessment of markers related to RPE and photoreceptor OS phagocytosis in the VS of (aged) MGs may be highly relevant in future studies on the pathophysiology of macular diseases. Moreover, proteins of the visual cycle critical for the clearance of all-*trans*-retinal from photoreceptors, such as the ATP-binding cassette transporter 4 (ABCA4) and the retinol dehydrogenase 8 (RDH8) enzymes, also showed enhanced abundance in the VS of the MG. In *Rdh8^−/−^Abca4^−/−^* mice, toxic byproducts of the visual cycle led to RPE/photoreceptor dystrophy with key features of AMD (44). The metabolism of these proteins in the VS of the MG may help us to understand disease-relevant visual cycle dynamics of the human central retina. In the current investigation, the limited sample size due to the restricted availability of biological samples from the MG may have prevented reaching the full potential of the proteomic analysis, as our filtering and selection parameters for valid peptides and proteins were set to the most stringent settings to allow higher accuracy in protein identification and quantification.

In summary, our data indicate that the MG, with its VS, may serve as a non-primate model to investigate the pathophysiology of human central retinal and macular diseases. As a rodent, it is phylogenetically similar to mice, which includes the advantages of relatively low cost and short lifespan, but combined with a retinal organization closer to that of larger animal models (e.g., pigs, cats, and dogs). Furthermore, the majority of commercially available antibodies developed for mice also work in the MG. Obviously, to fully represent the human central retina, the presence of a fovea would be to strive for. However, non-human primates that do have a macula with a fovea are problematic to use in research in larger numbers due to their high cost, slow disease progression, difficulty with genetic manipulations, and ethical concerns (45). Importantly, the first CRISPR/Cas9-mediated mutant MG models have been generated (46, 47), indicating the availability of genetic manipulation. Further studies on such mutants will show whether the MG may also serve as a disease model in conditions of retinal dystrophy such as AMD and Stargardt disease.

## Data Availability

All raw files and search results including spectral library, search parameters and search results have been submitted to the Massive and ProteomeXchange databases and made fully accessible to the scientific community under the project IDs: MassIVE MSV000097218 and PXD061216.
