# Supplementary material for: Exploration of the visual streak of the Mongolian gerbil as a model for the human central retina
Source: Front Med (Lausanne). 2025 Apr 22;12:1562437. doi: 10.3389/fmed.2025.1562437 (PMC12086270; doi:10.3389/fmed.2025.1562437)
Supplement: Supplementary file 2 [file Data_Sheet_1.docx]

Supplementary Material


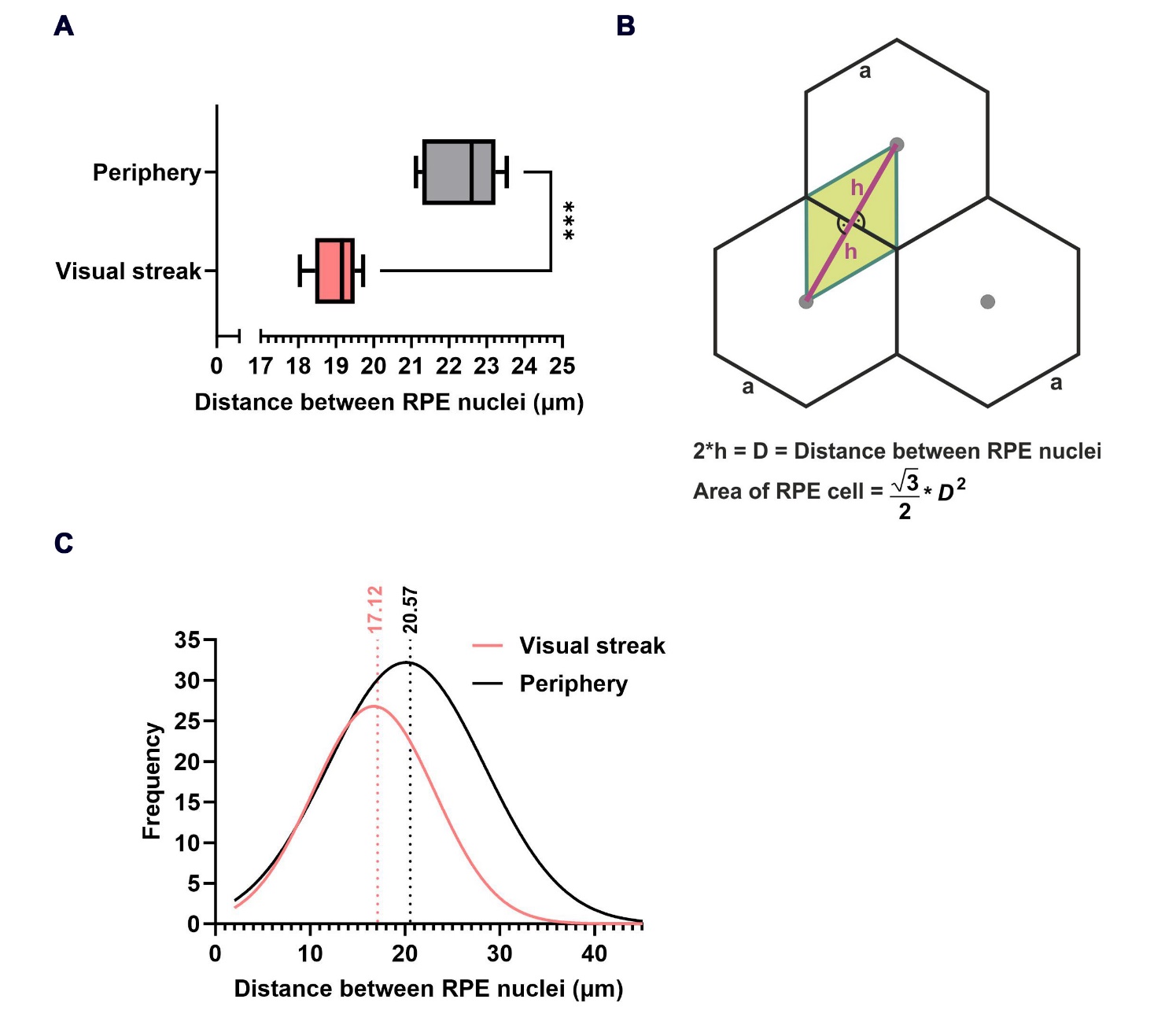


**Figure S1. Parameters for the analysis of RPE cell areas. (A)** Quantitative evaluation of the distances between the RPE nuclei (box-and-whisker-plot) in the VS (n = 5) compared to the peripheral retina. Boxes: 25%-75% quantile range, whiskers: 5% and 95% quantiles, central line: median. **(B)** Mathematical model based on the simplifying assumption of a hexagonal RPE monolayer used to calculate RPE cell area based on the distances between nuclei. D = distance between nuclei; h = height; a = side. **(C)** Histogram for the frequency distribution from all 454 RPE nuclei distances in the VS and 724 in the peripheral retina. Values lower than half of the median of the frequency distributions were considered artifacts by binucleate cells, and values higher than twice the median were considered artifacts by skipped nuclei in the retinal sections. Highlighted values represent the medians of the respective regions.


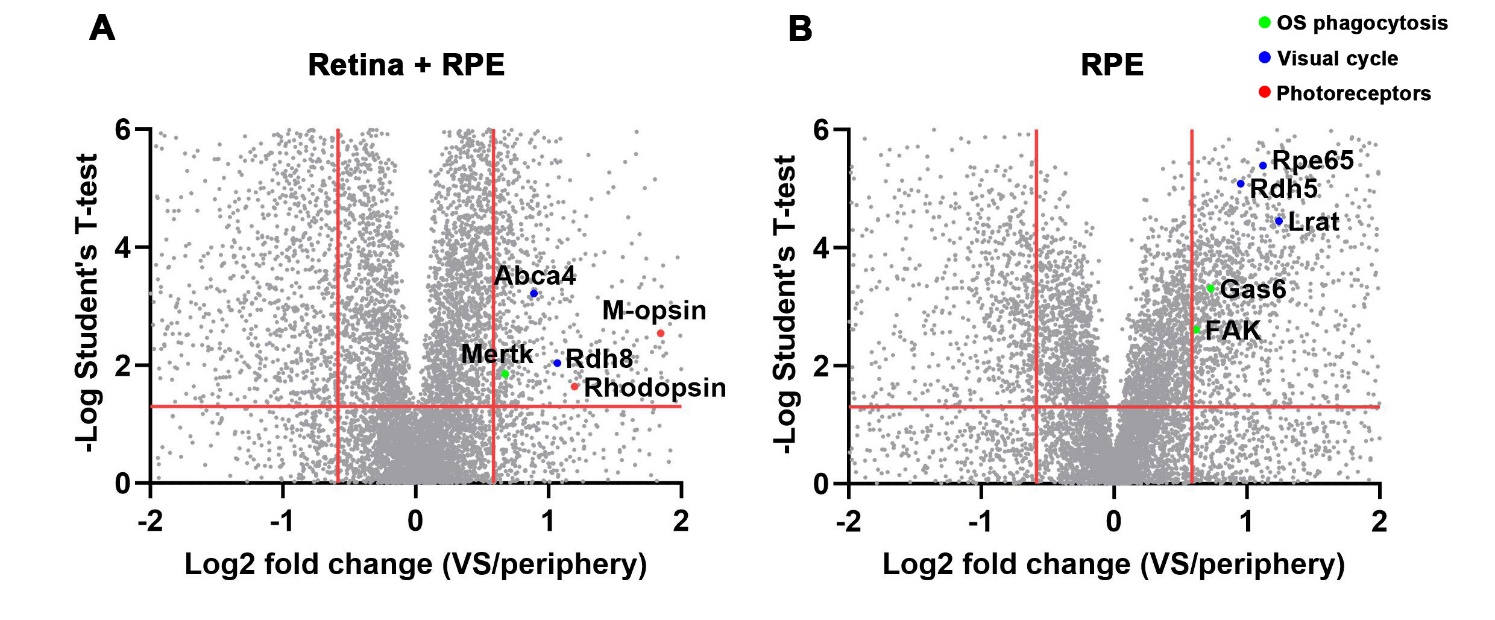


**Figure S2. Proteomic mass spectrometry-based differential expression analysis of MGs in the VS compared to the peripheral retina.** Volcano plots showing significant differential abundant proteins in **(A)** the interface of the retina + RPE and **(B)** in the isolated RPE by quantitative proteomic analysis. The p-value was plotted on the y-axis as -log_10_ and the relative abundance ratio of VS/periphery on the x-axis as a log_2_ fold change. Red vertical lines indicate the cutoff for a significant enrichment- or depletion of protein abundance of 1.5-fold. The red horizontal line indicates a p-value of 0.05. VS = Visual streak.

**Supplementary Spreadsheet 1. List of proteins identified and quantified across all samples.**
